# Construction Notes

**Published:** 1896-10-10

**Authors:** 


### CONSTRUCTION NOTES.

### The Hope Hospital for Langholm.

The memorial stone of the Thomas Hope Hospital was laid by Miss Jane Hope, of New York, who is sister to the donor, Mr. Thomas Hope, of New York, who left upwards of £100,000 for the purpose of building and endowing the hospital. The building, which is far advanced towards completion, is intended to form a suitable as well as useful and ornamental memorial of its donor. It has a breadth of about 200 feet, while the other way, inclusive of lodge, the measurement is 144 feet. The architects are Messrs. Woodd and Ainstie, of London. The material used for the construction of the hospital is the white freestone of the district. The walls, which are thick and massive, are of rock-faced work, with finely proportioned gables, pediments, and circular dormer windows. The main entrance is from Market Place, whence the hospital is reached by the main corridor, which is seven feet wide. This leads, in the first place, through the administrative block, which contains doctors' rooms communicating with the operating-room, and lavatory adjoining. These are on the right; on the left is the visitors' room, &c. Further on is a passage leading to the mortuary, and then on the left of the main building is the woman's section, which has a corridor six feet wide, off which there are two single-bedded wards, convalescent-room, nurses' room, and a five-bedded ward. A lavatory wing contains bath-room, &c. On the right of the principal corridor there is a dispensary, with a separate entrance for dispensing to outside patients. Near this part of the building is the matron's-room, which separates the men's and women's sections. Passing through swing glass doors the men's section is entered, which contains essentially the same accommodation as the women's. On the left is a staircase leading to servant's bed-rooms on first floor, and to tank-room, box-room, &c., higher up. There is a kitchen wing to the right of the corridor, which contains excellent accommodation, and here also are to be found heating furnaces, stores, pantries, &c. There is a laundry adjoining. The building throughout is being erected in the most substantial manner. The contractors are Messrs. Thomas Telford, of Langholm.

### New Infirmary at Bridgnorth.

The new infirmary has a pleasing appearance, and is built with brick and tile, the architect being Mr. Edward C. H. Maidman, of Albyn Place, Edinburgh, the contractor being Mr. Hughes, builder, of Birmingham. All unnecessary ornamentation has been avoided, the one object of the committee having been to secure hygienic perfection in a building so completely planned as to be capable of being worked by a small staff. There are two very large wards, and one special ward containing eighteen beds. On entering the building the out-patients' waiting-room is first arrived at, a spacious room, opening on the consulting-room, and from thence to the dispensary. Here is a room for the house surgeon, and the matron's sitting-room. The laundry is situated a few yards from the general infirmary, beyond which is the mortuary. There is a special ward for surgical cases, and directly opposite this is the operating-room, facing north. From the nurse's duty-room a good view is obtained of both wards. The lavatory arrangement for each ward is complete, and entirely separated from the rooms, by distinct corridors with cross ventilation. There are incandescent burners throughout the institution, outside of which is a verandah for convalescents. The wards are named after the respective donors. The cost of building the infirmary is estimated at £5,000.

### Newport Infectious Diseases Hospital.

This hospital, erected by the Newport Corporation, has been formally opened. It is situated 260 feet above ordnance level, and commands a fine view. The area of the site is a little over five and a-quarter acres. The buildings consist of an administration block, a pair of fever pavilions, isolation block, laundry, porter's lodge, and a mortuary. All of the blocks, with the exception of the laundry, are built of brick, with Bath stone dressings. The administration block is furnished with every convenience, and has an imposing appearance. Each of the fever pavilions contains two wards, 36 feet long, 26 feet wide, and 13 feet high. Each ward is laid out with six beds, consequently 2,006 cubic feet of air for each patient is provided. The ventilation is most complete, and the heating will be provided by Boyd's patent down draught stove. The ward linen is kept in ventilated compartments. The nurses have a room in the centre of each pavillion, with fixed windows on either side, commanding all the beds. In the isolation block the wards are somewhat similar in arrangement, but adapted to the Local Government Board requirements for special use. The laundry, disinfecting room, and mortuary are fitted up with every modern improvement. The total cost will be just under £14,000. Messrs. A. S. Morgan and Co., of Newport, were the contractors for the work, the designs of which were executed by the borough engineer.

